# Current-voltage characteristics of nanoplatelet-based conductive nanocomposites

**DOI:** 10.1186/1556-276X-9-369

**Published:** 2014-07-29

**Authors:** Amirhossein Biabangard Oskouyi, Uttandaraman Sundararaj, Pierre Mertiny

**Affiliations:** 1 University of Alberta, 4-9 Mechanical Engineering Building, Edmonton, Alberta T6G 2G8, Canada; 2 Department of Chemical and Petroleum Engineering, University of Calgary, Alberta T2N 1 N4, Canada

**Keywords:** Nanocomposites, Conductive nanoplatelets, Current-voltage characteristics

## Abstract

In this study, a numerical modeling approach was used to investigate the current-voltage behavior of conductive nanoplatelet-based nanocomposites. A three-dimensional continuum Monte Carlo model was employed to randomly disperse the nanoplatelets in a cubic representative volume element. A nonlinear finite element-based model was developed to evaluate the electrical behavior of the nanocomposite for different levels of the applied electric field. Also, the effect of filler loading on nonlinear conductivity behavior of nanocomposites was investigated. The validity of the developed model was verified through qualitative comparison of the simulation results with results obtained from experimental works.

## Background

In recent years, the nonlinear electrical conductivity behavior of nanoparticle-modified polymers has received considerable attention by researchers, and several studies have been carried out to investigate the current-voltage characteristics of conductive nanocomposites. Even though several studies investigated the nonohmic conductivity behavior of insulator polymers filled with conductive spherical and stick-like inclusions [1-5], to the best of the authors' knowledge, all of the research in this field has been limited to experimental works. Experimental research devoted to the electric properties of nanoplatelet-based nanocomposites investigated the electrical conductivity of polymers with exfoliated graphite sheets with sizes varying from a few microns to several hundreds of a micron [6-10], which allows for only limited prediction of the conductivity behavior of nanocomposites with submicron size inclusions.

These limitations motivated the present authors to conduct a numerical study to investigate the current-voltage behavior of polymers made electrically conductive through the uniform dispersion of conductive nanoplatelets. Specifically, the nonlinear electrical characteristics of conductive nanoplatelet-based nanocomposites were investigated in the present study. Three-dimensional continuum Monte Carlo modeling was employed to simulate electrically conductive nanocomposites. To evaluate the electrical properties, the conductive nanoplatelets were assumed to create resistor networks inside a representative volume element (RVE), which was modeled using a three-dimensional nonlinear finite element approach. In this manner, the effect of the voltage level on the nanocomposite electrical behavior such as electrical resistivity was investigated.

## Methods

### Monte Carlo modeling

**Figure 1:**
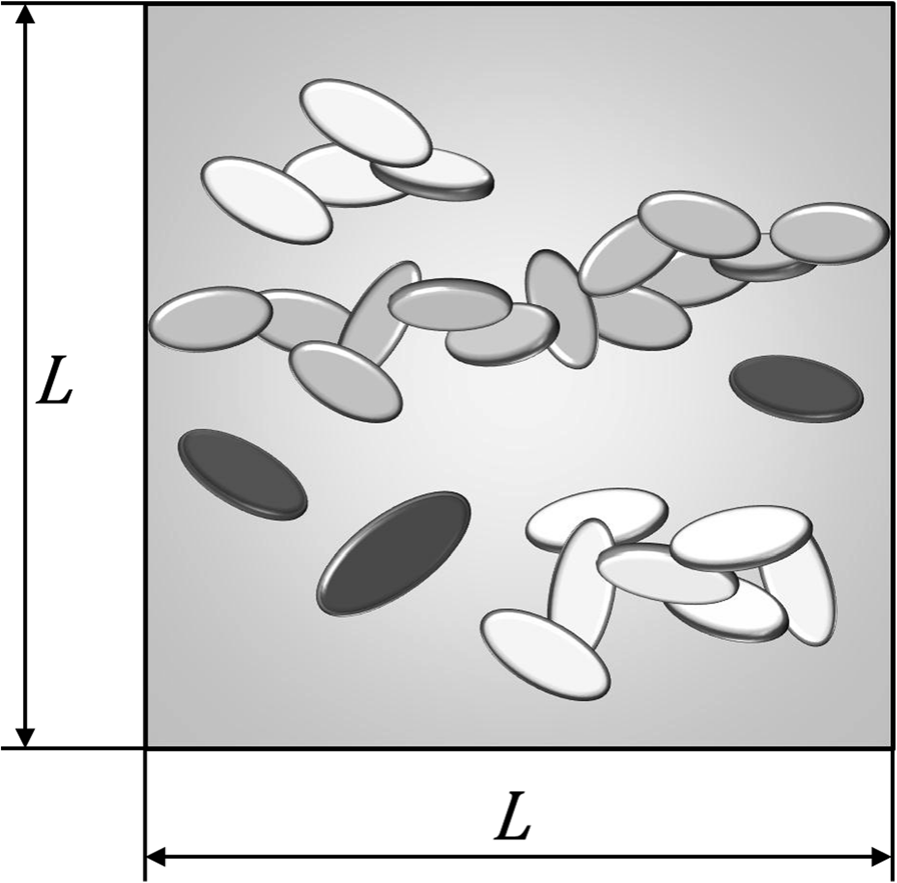
Schematic of a representative volume element illustrating nanoplatelets (black), clusters (white), and percolation network (gray).

**Figure 2:**
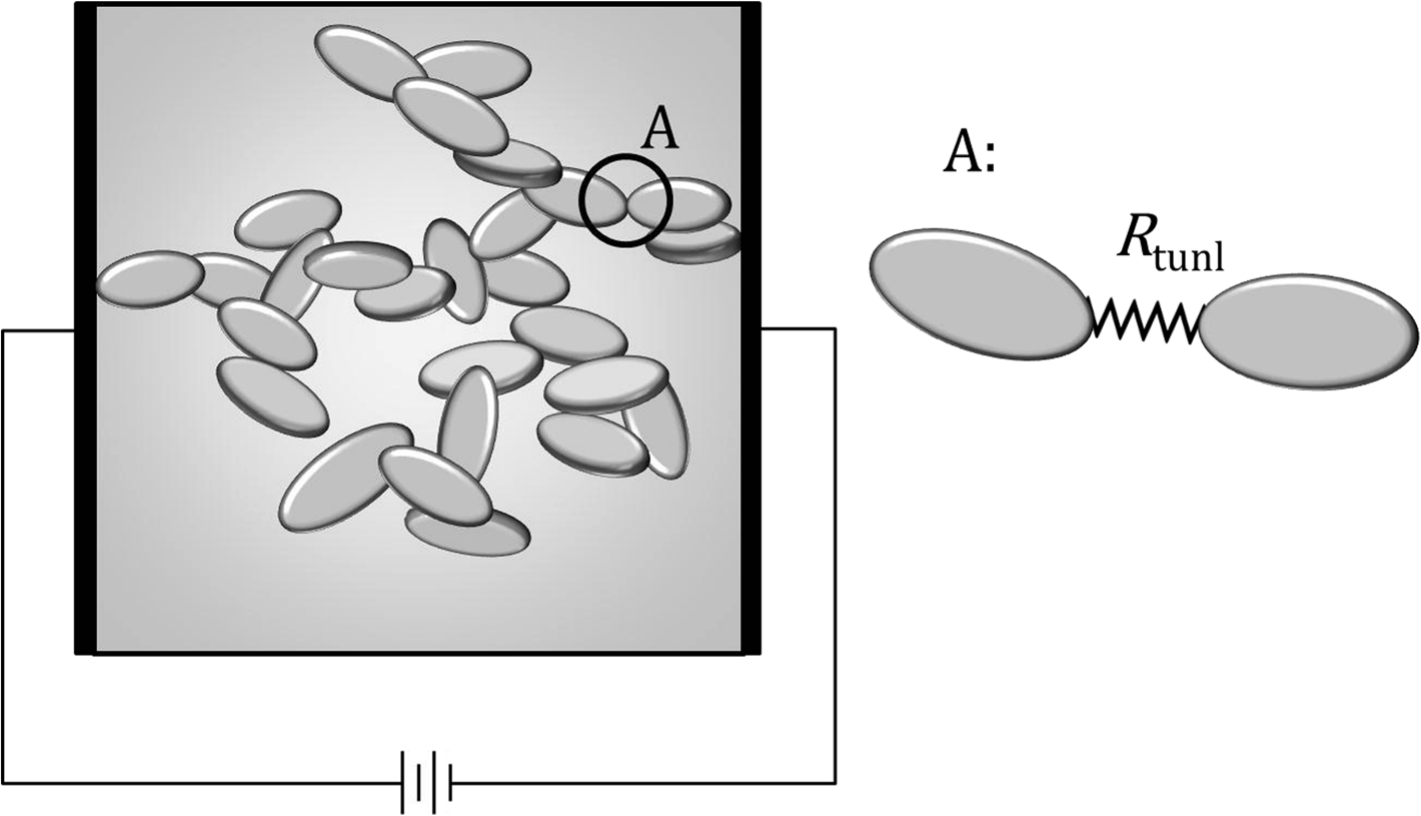
Illustration of tunneling resistors.

Theoretically, a nanocomposite is rendered electrically conductive by inclusions dispersed inside the polymer that form a conductive path through which an electrical current can pass. Such a path is usually termed a percolation network. Figure 1 illustrates the conductivity mechanism of an insulator polymer made conductive through the formation of a percolation network. In this figure, elements in black, white, and gray color indicate nanoplatelets that are individually dispersed, belong to an electrically connected cluster, or form a percolation network inside the RVE, respectively. Quantum tunneling of electrons through the insulator matrix is the dominant mechanism in the electric behavior of conductive nanocomposites. Figure 2 illustrates the concept of a tunneling resistor for simulating electron tunneling through an insulator matrix and its role in the formation of a percolation network.

Electron tunneling through a potential barrier exhibits different behaviors for different voltage levels, and thus, the percolation behavior of a polymer reinforced by conductive particles is governed by the level of the applied voltage. In a low voltage range (*eV* ≈ 0), the tunneling resistivity is approximately proportional to the insulator thickness, that is, the tunneling resistivity shows ohmic behavior [11]. For higher voltages, however, the tunneling resistance is no longer constant for a given insulator thickness, and it has been shown to depend on the applied voltage level. It was derived by Simmons [11] that the electrical current density passing through an insulator is given by

(1) $$J=J_{0}\left\{\bar{\lambda }\exp\left(-A{\bar{\lambda }}^{\frac{1}{2}}\right)\right)\left(-\left(\bar{\lambda }+\mathrm{eV}\right)\exp\left[-A\left(-A{\bar{\lambda }}^{\frac{1}{2}}\right)\right]\right\}$$

where

*J*_0_ = *e*/2*πh*(*β*Δ*s*)^2^ and $A=\left(4\pi \beta \Delta s/h\right){\left(2m\right)}^{\frac{1}{2}}$

Considering Equation 1, even for comparatively low voltage levels, the current density passing through the insulator matrix is nonlinearly dependent on the electric field. For vanishing voltages, when *eV* ≈ 0, Equation 1 can be simplified as [11]

(2) $$J=\frac{e^{2}\sqrt{2\mathrm{m\lambda}}}{h^{2}\;}V\exp\left(-\frac{4\mathrm{\pi d}}{h}\sqrt{2\mathrm{m\lambda}}\right)$$

**Figure 3:**
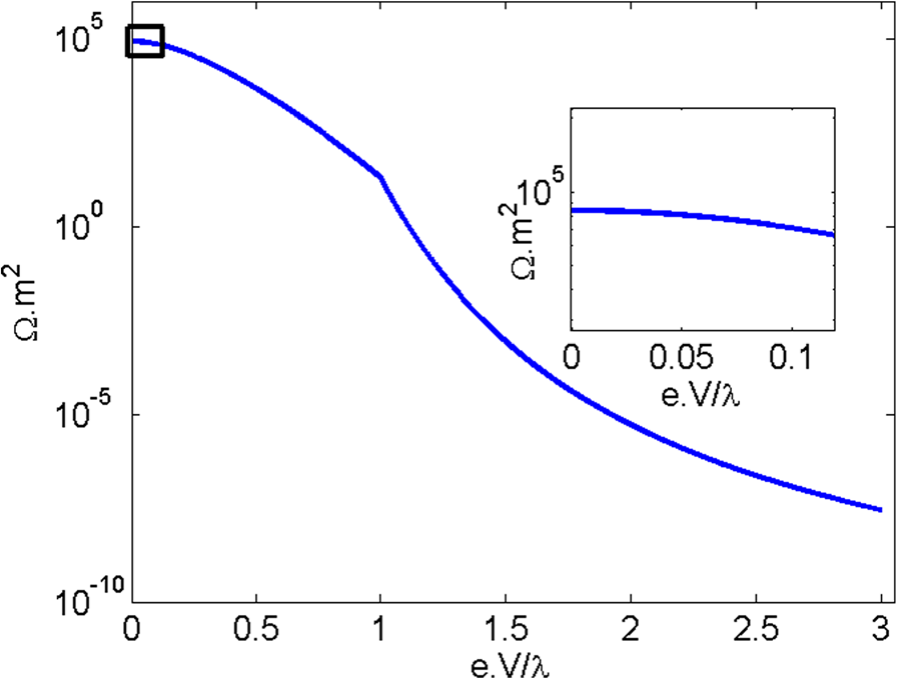
**Polymer resistivity per unit area versus normalized voltage.** The inset shows approximately ohmic behavior for low electric fields.

The numerical evaluation of Equation 1 given in Figure 3 shows the resistivity per unit area of polymer with 4-nm thickness and a quantum tunneling barrier height *λ* of 1 eV, with respect to the normalized voltage eV/*λ*. This graph indicates that a polymer only exhibits close to ohmic behavior when subjected to low electric fields, that is, the resistivity of the polymer is approximately constant in a small region near the ordinate axis (see inset in Figure 3), permitting the use of the linear approximation provided by Equation 2.

**Figure 4:**
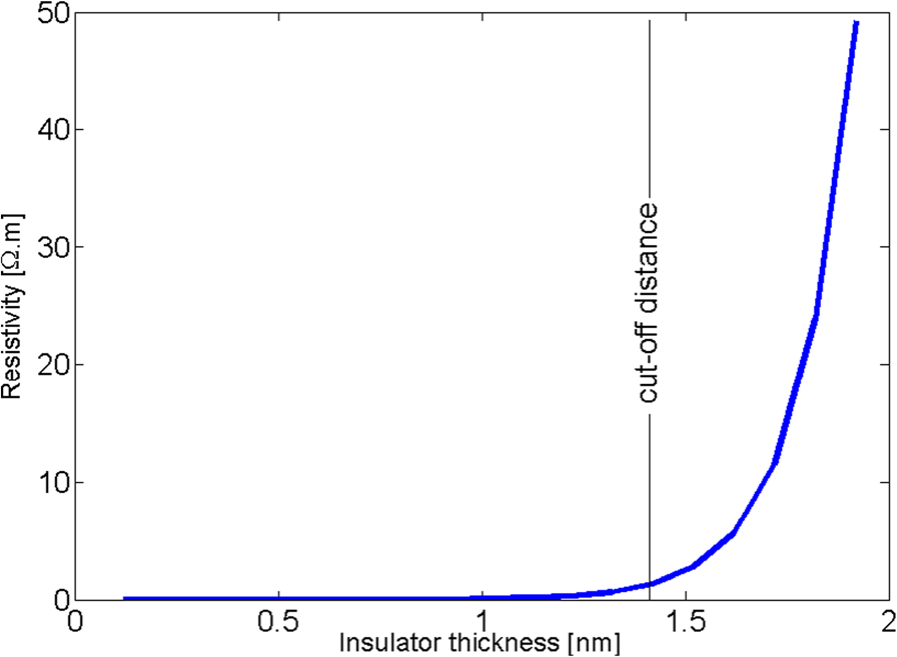
Tunneling resistivity versus insulator thickness.

In this study, a rectangular potential barrier was assumed to model the electrical behavior of the tunneling resistor. Tunneling resistivity is numerically evaluated for *λ* = 0.5 ev employing Equation 2 and illustrated in Figure 4. The tunneling resistance is drastically dependent on the insulator thickness, that is, tunneling resistance is sharply increasing as the insulator thickness is increasing. A cutoff distance can therefore be approximated at which tunneling resistors with length greater than this threshold do not appreciably contribute toward the overall conductivity of the nanocomposite. In [12] and [13], the cutoff distance was assumed to be 1.0 and 1.4 nm, respectively. It is expected that the resistivity of the insulator film is decreasing as the electrical field is increasing; so, when dealing with higher voltage levels, tunneling resistors with length greater than these cutoff distances may play a role in the nanocomposite conductivity. Hence, it was conservatively assumed in this study that tunneling resistors with length less than 4 nm contribute toward the nanocomposite conductivity.

In the first step of this work, a three-dimensional continuum percolation model based on Monte Carlo simulation was used to study the percolation behavior of an insulator matrix reinforced with conductive nanoplatelet fillers. Additional details on this modeling approach can be found in an earlier publication [14]. In the simulation, circular nanoplatelets are randomly generated and added to the RVE. The shortest distance between adjacent particles is calculated, and particles with distance between them shorter than the cutoff distance are grouped into clusters. The formation of a cluster connecting two parallel faces of the RVE is considered the formation of a percolation network that allows electric current to pass through the RVE, rendering it conductive.

### Finite element modeling

To study the electrical properties of nanocomposites, in particular their conductivity behavior, the employed modeling approach further involved the creation of a nonlinear three-dimensional finite element resistor network. Considering the excellent conductivity of the considered nanoplatelets (e.g. *σ* = 10^8^ S/m for graphene), the electrical potential drop across the nanoplatelets was neglected. The tunneling resistors were modeled as nonohmic, which resistivity is governed by Equation 1. Employing appropriate finite element formulations, the governing equation of an electrical resistor can be written as

(3) $$\left\{\begin{array}{c} I_{\mathrm{ij}}\\ -I_{\mathrm{ij}} \end{array}\right\}=\;\left[\begin{array}{l} k_{\mathrm{ij}}\;-k_{\mathrm{ij}}\\ -k_{\mathrm{ij}}\;k_{\mathrm{ij}} \end{array},\;\right]\;\left\{\begin{array}{l} V_{i}\\ V_{j} \end{array}\right\}$$

where *I*_*ij*_ is the electrical current passing between the *ith* and *jth* node; *k*_*ij*_ is the conductance of the resistor between nodes *i* and *j*; and *V*_*i*_ is the voltage of the *ith* node measured with respect to a node connected to ground. The system of the nonlinear equations governing the electrical behavior of the nanocomposite was obtained by assembling the governing equations for the individual elements. The resulting nonlinear system of equations was solved employing an iterative method.

## Results and discussion

### Modeling results

**Figure 5:**
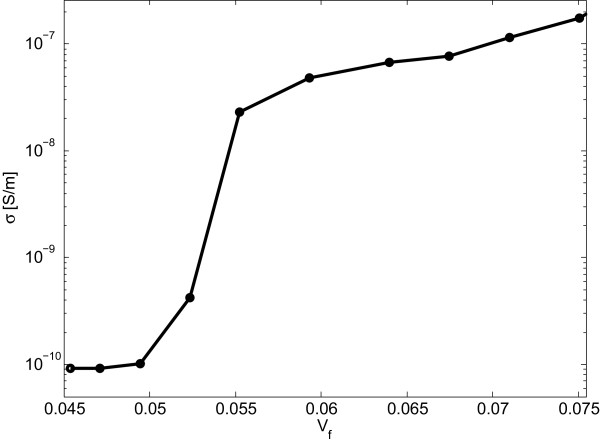
Conductivity of nanocomposite with respect to filler loading of conductive nanodisks with diameter of 100 nm.

**Figure 6:**
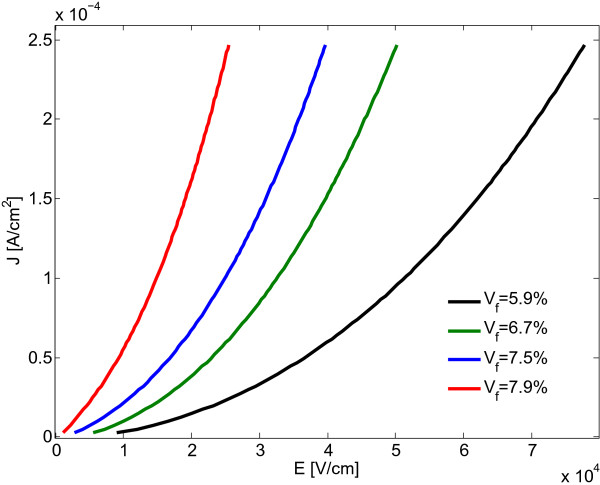
Electric current density of nanocomposites with 100-nm-diameter nanoplatelets versus the applied electrical field.

The developed model was employed to investigate the electrical behavior of a polymer with *λ* = 0.5 ev made conductive through the uniform dispersion of conductive circular nanoplatelets with a diameter of 100 nm. In the simulations, the size of the RVE was chosen to be nine times the diameter of the nanodisks, which was ascertained to be large enough to minimize finite size effects. In an earlier study [15], the authors showed that the Monte Carlo simulation results are no longer appreciably RVE-size dependent when the RVE size is about eight times the sum of *2R* + *d*_*t*_, where *R* and *d*_*t*_ are the radius of the nanoplatelets and tunneling distance, respectively.The graph in Figure 5 depicts the effect of filler loading on nanocomposite conductivity. As expected, a critical volume fraction indicated by a sharp increase in nanocomposite conductivity, i.e., the percolation threshold, can be inferred from the graph.In the following, electric current densities passing through the nanocomposite RVE were computed for different electric field levels and filler volume fractions. As illustrated by Figure 6, the current density versus voltage curves were found to be nonlinear. The depicted electrical behavior of the conductive nanocomposite is thus clearly governed by the applied voltage in a nonohmic manner, which, as mentioned above, matches the expectation for a conductive nanocomposite at higher electric field levels.

**Figure 7:**
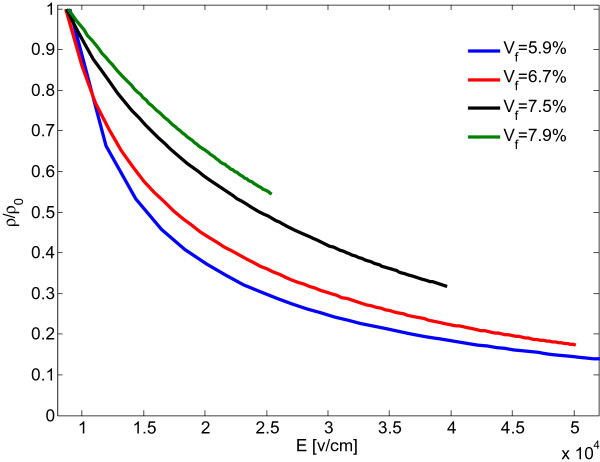
Normalized resistivity of nanocomposites with 100-nm nanodisks as a function of the applied electrical field.

Figure 7 shows the variation of resistivity as a function of the applied electric field *E* in order to compare the nonohmic behavior for nanocomposites with different filler loadings. Note that resistivity values were normalized with respect to a reference resistivity measured at *E* = 0.8 V/cm. The results as displayed in Figure 7 indicate that the magnitude of the applied electric field plays an important role in the conductivity of nanoplatelet-based nanocomposites. The employed modeling approach predicts nanocomposite resistivity to be a nonlinear function of the applied voltage. Clearly, the nanocomposites exhibit nonohmic behavior where the resistivity decreases with increasing voltage. Interestingly, the results in Figure 7 also indicate a reduced drop in resistivity and decreased nonohmic behavior for nanocomposites with higher filler volume fraction, that is, nanocomposites with higher filler loadings are less sensitive to the applied electric field level.

### Comparison with experimental data

**Figure 8:**
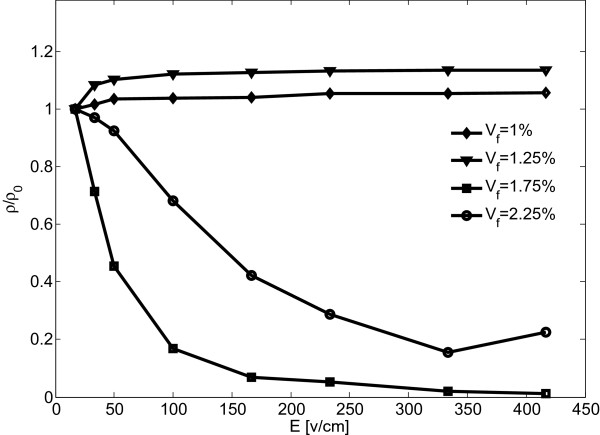
Normalized resistivity data versus applied electrical field from experiments with nanographene/epoxy samples.

To corroborate the simulation results, conductive epoxy nanocomposite samples were produced by in situ polymerization and their electrical behavior assessed as illustrated by Figure 8. Bisphenol-A epoxy resin and non-MDA polyamine curing agent (EPON 826 and EPIKURE 9551, by Hexion Specialty Chemicals, Columbus, Ohio, USA) were used for the fabrication of samples that were made electrically conductive by dispersing graphene nanoplatelets (xGnP-M-25, by XG Sciences, Lansing, Michigan, USA).

Graphene nanoplatelets were dispersed in acetone by sonication using a probe sonicater in an ice bath. In the following, epoxy was added to the mixture and sonication was repeated. The solvent was evaporated by heating the mixture on a magnetic stir plate and stirring with a Teflon-coated magnet. Remaining acetone was removed by using a vacuum chamber. The curing agent was added to the mixture and mixed with a high-speed mechanical shear mixer. The mixture was again degassed using the vacuum chamber and subsequently poured into a mold. A 2-h cure cycle was then performed at 120°C. Resulting samples were machined into circular disks with 30-mm diameter and 3-mm thickness. The sample volume resistivities were measured at different applied voltages employing a Keithley 6517A electrometer connected to a Keithley test fixture (Keithley Instruments, Cleveland, Ohio, USA).Data in Figure 8 depicting the resistivity behavior of the epoxy nanocomposite samples was normalized with respect to the resistivity measured at an applied voltage of 10 V. Samples with 1 and 1.25% graphene volume fraction exhibited high resistivity levels indicating a filler loading below the percolation threshold. For higher graphene volume fractions of 1.75 and 2.25%, measurements indicated that percolation was achieved, and resistivity was found to decrease with the increase of the applied electric field. As predicted by the preceding modeling work, sample resistivity was found to be less sensitive to the applied electrical field for higher filler loadings. Hence, modeling and simulation results are qualitatively in good agreement, indicating the validity of the assumptions undertaken for the numerical modeling. However, the data presented in Figures 7 and 8 also signify that further studies are warranted to establish a quantitative agreement between numerical and experimental results.

### Characterization of resistivity behavior

Gorrasi et al. [5] and Liu et al. [16] showed that the resistivity of carbon nanotube-based nanocomposites as a function of the electric power *P = V × I* can be described by an exponential expression:

(4) $$\rho =r\;P^{\alpha }$$

where *α* is an index which generally varies between −1 and 0. The value of *α* is indicative of the nonlinearity of the current-voltage relationship, i.e., *α* = 0 corresponds to ohmic behavior, and *α* decreases with increasing nonlinearity of the current-voltage curve; *r* is a parameter relating to the resistivity of the nanocomposite when the electrical power passing through the sample is 1 W [16].

**Figure 9:**
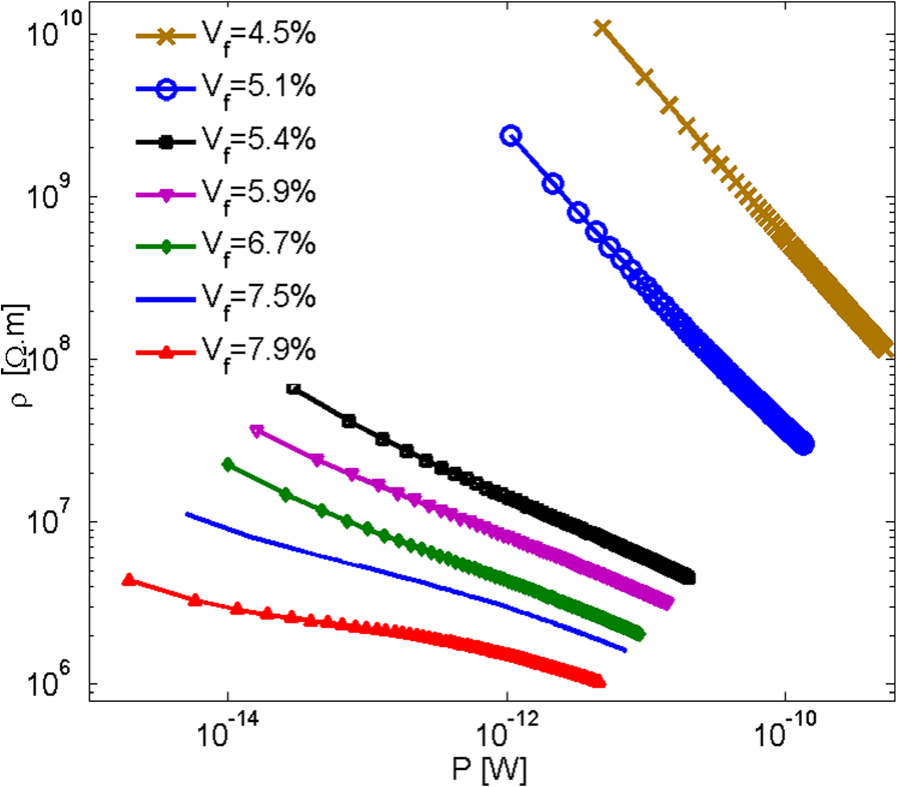
Resistivity of nanocomposites with 100-nm circular nanoplatelets as a function of electric power.

**Figure 10:**
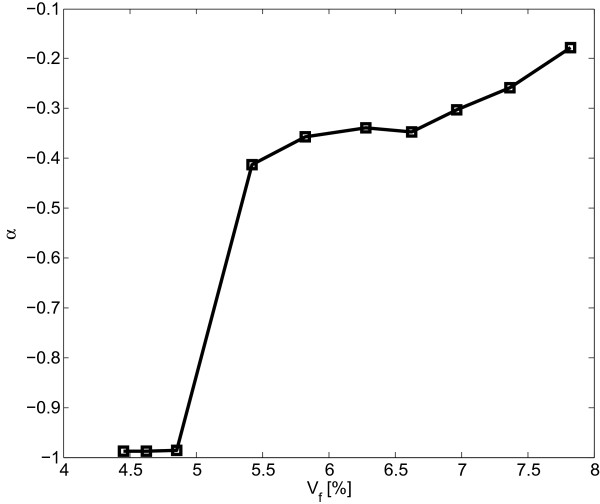
**Value of *α *** **as a function of filler volume fraction for nanocomposites with 100-nm circular nanoplatelets.**

Computed nanocomposite resistivities are displayed as a function of the electric power in the graph in Figure 9. Data obeying Equation 4 appear in the form of straight lines owing to the graph's logarithmic scale. As shown in Figure 9, the slope of the lines decreases as the nonlinearity is decreasing with increasing filler loading. The values of *α* as a function of filler volume fraction are provided in Figure 10. It is shown that *α* values are increasing with rising filler volume fraction. A discontinuity in *α* values can be observed in this graph for filler volume fractions of about 5%, which is associated with the percolation volume fraction. The behavior of data simulated herein is qualitatively congruent with results reported in [5] for carbon nanotube nanocomposites.

## Conclusions

In this study, the current-voltage behavior of conductive nanoplatelet-based nanocomposites was investigated. To this end, a numerical modeling approach was developed. The simulations predicted the resistivity of nanoplatelet-based nanocomposites to be strongly affected by the applied electric field. The nanocomposites exhibit nonohmic behavior, that is, resistivity is a nonlinear function of the applied electric field. Further, nanocomposite resistivity was ascertained to decrease with increasing voltage, while the degree of nonlinear behavior was found to decline with rising filler volume fraction. A good qualitative agreement was observed between simulations and experimental data, the latter of which was obtained employing measurements on nanographene/epoxy nanocomposites. The qualitative agreement between numerical and experimental studies encourages conducting a more comprehensive study to establish a quantitative agreement. The analysis further revealed that nanocomposite resistivity as a function of electrical power can be described by an exponential relation, where the exponent is a measure of the deviation from nonohmic behavior of the conductive nanocomposite.

## Nomenclature

*d*_*t*_, tunneling distance

*e*, electron charge

*E*, applied electric field

*h*, Planck's constant

*I*, electrical current

*J*, quantum tunneling current density

*k*, conductance of tunneling resistor

*L*, dimension of representative volume element

*m*, electron mass

*P*, electric power

*r*, resistivity parameter

*R*, radius of circular nanoplatelets

*s*_*1*_*, s*_*2*_, limits of barrier at Fermi level

Δ*s = s*_2_ *− s*_1_

*V*, voltage across insulator

*V*_*f,*_ filler volume fraction

*α*, nonlinearity factor

*β*, correction factor

*λ*, height of barrier for quantum tunneling 

$\bar{\lambda },$ mean barrier height for quantum tunneling

*ρ*, electrical resistivity

*σ*, electrical conductivity

## Competing interests

The authors declare that they have no competing interests.

## Authors' contribution

All authors made equally valuable contributions to this paper. All authors read and approved the final manuscript.
